# Supplementary material for: Effects of MWCNTs on Char Layer Structure and Physicochemical Reaction in Ethylene Propylene Diene Monomer Insulators
Source: Polymers (Basel). 2022 Jul 26;14(15):3016. doi: 10.3390/polym14153016 (PMC9330200; doi:10.3390/polym14153016)
Supplement: Supplementary file 1 [file polymers-14-03016-s001.zip › polymers-1821230-supplementary.pdf]

Supporting Information

Effects of MWCNTs on char layer structure and ablative resistance of EPDM-based insulators

Zhiheng Chen[a], Shida Han[a], Yuan Ji[a], Hong Wu[a],, Shaoyun Guo[a], Ning Yan[b],**, Hongyan Li[b]*

[a]*The State Key Laboratory of Polymer Materials Engineering, Polymer Research Institute of Sichuan University, Chengdu 610065, China*

[b]*Xi'an Modern Chemistry Research Institute, Shaanxi, Xi'an, 710065, China*

1. The dispersion of MWCNTs in EPDM-based insulators

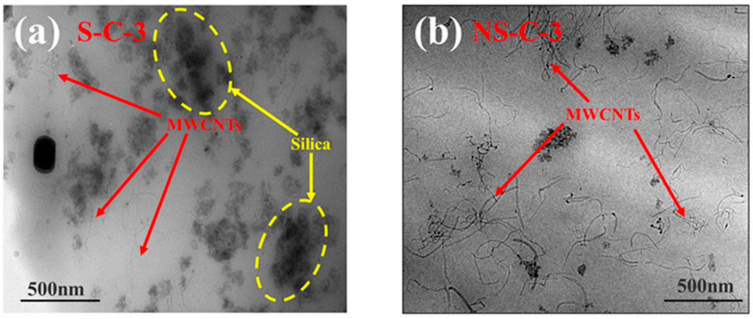

Figure S1. The dispersion of 3 phr MWCNTs in EPDM-based insulators with (a) and without silica (b).

2. TGA of MWCNTs, silica and EPDM-based insulators

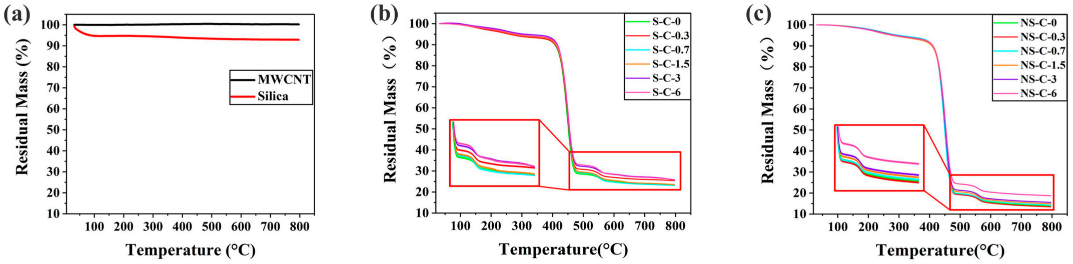

Figure S2. Thermal stability of MWCNTs (a) and the effects of MWCNTs content on the residual mass of insulators (group S (b), group NS (c)) after thermogravimetric analysis.

3. SEM image and pore diameter statistics of char layer surface in group NS

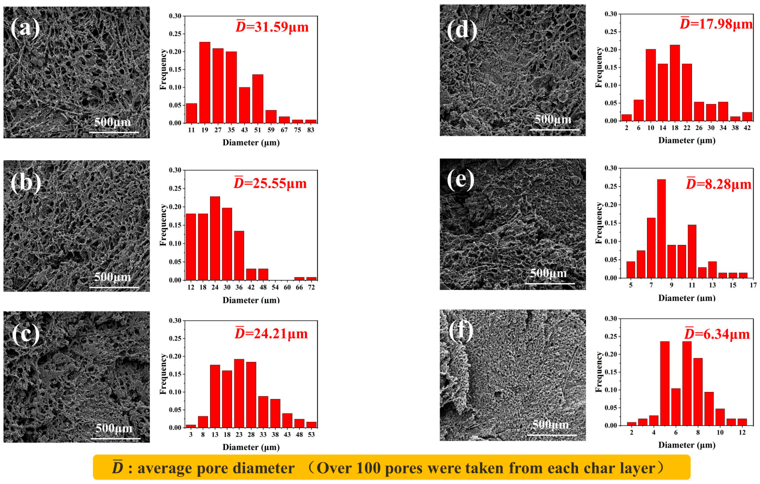

Figure S3. Effects of MWCNTs content on the average pore diameter of char layer surface of NS-C-0 (a), NS-C-0.3 (b), NS-C-0.7 (c), NS-C-1.5 (d), NS-C-3 (e), NS-C-6 (f).

4. SEM image and EDS results of char layer section in group NS

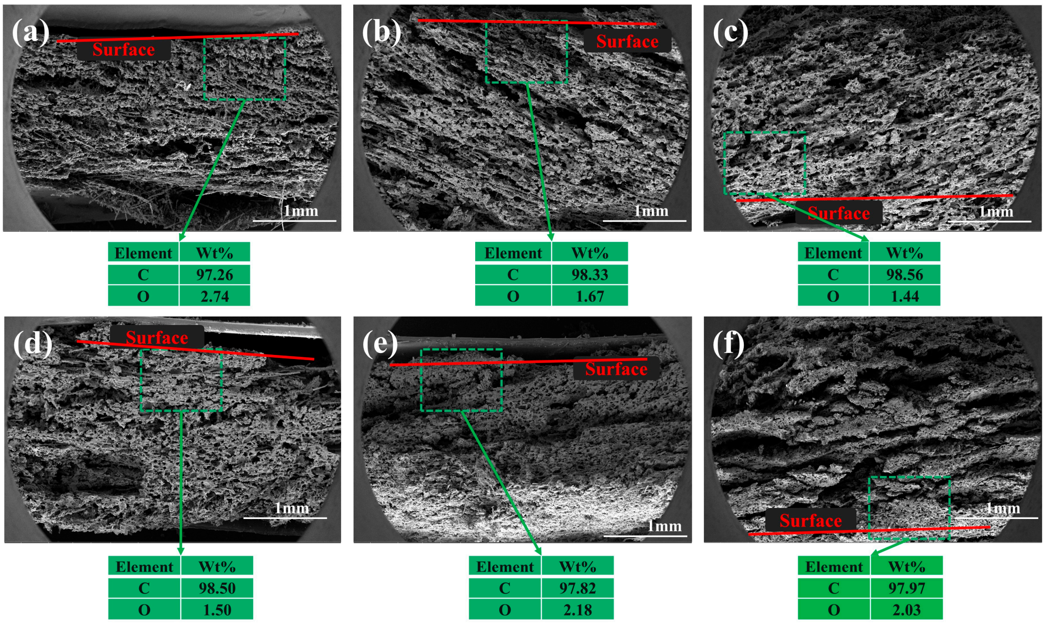

Figure S4. Effects of MWCNTs content on section structure and the element content of char layer: NS-C-0 (a), NS-C-0.3 (b), NS-C-0.7 (c), NS-C-1.5 (d), NS-C-3 (e), NS-C-6 (f).

5. SEM image and pore diameter statistics of char layer surface in group S

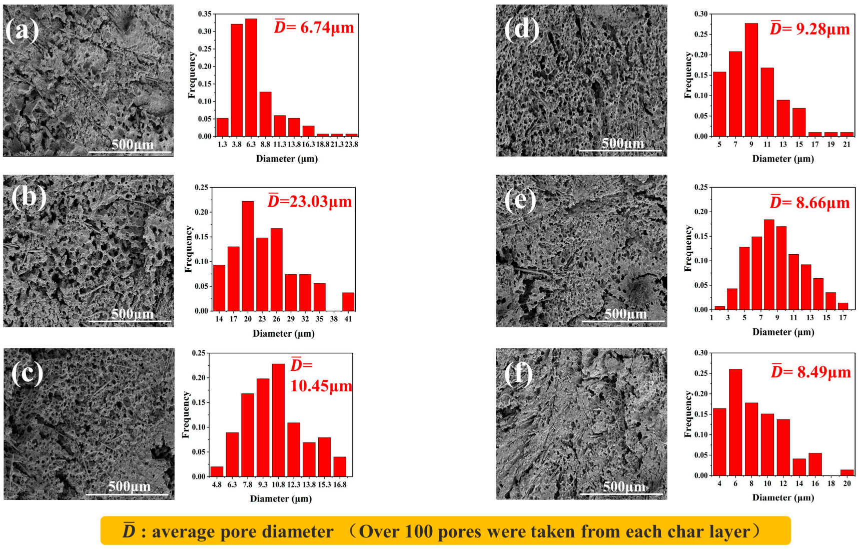

Figure S5. Effects of MWCNTs content on the average pore diameter of char layer surface of S-C-0 (a), S-C-0.3 (b), S-C-0.7 (c), S-C-1.5 (d), S-C-3 (e), S-C-6 (f).

6. SEM image and EDS results of char layer section in group S

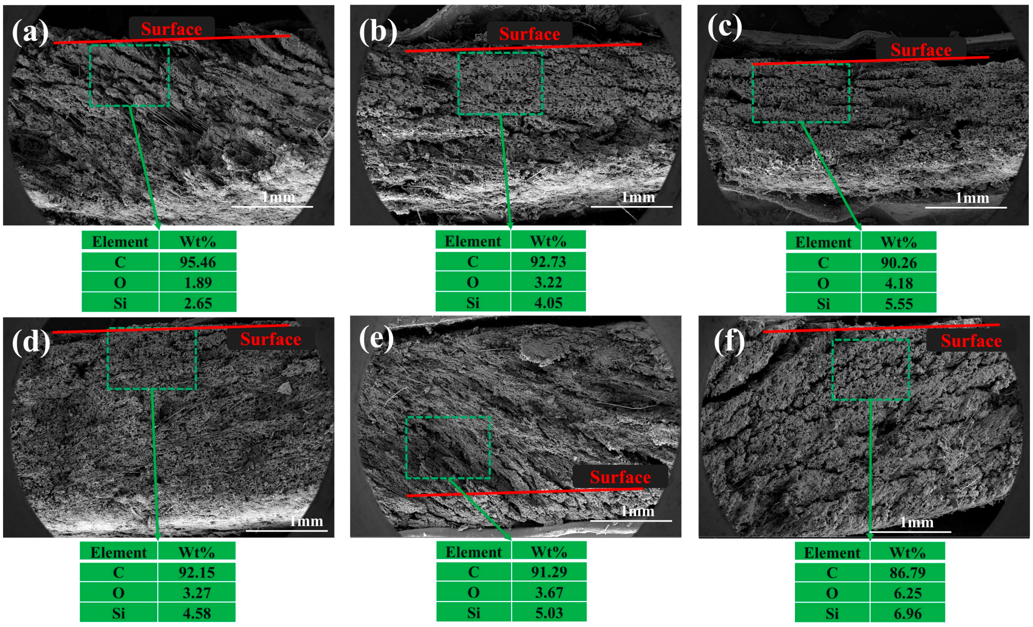

Figure S6. Effects of MWCNTs content on section structure and the element content of char layer: S-C-0 (a), S-C-0.3 (b), S-C-0.7 (c), S-C-1.5 (d), S-C-3 (e), S-C-6 (f)

7. Magnifying SEM image of char layer cross-section

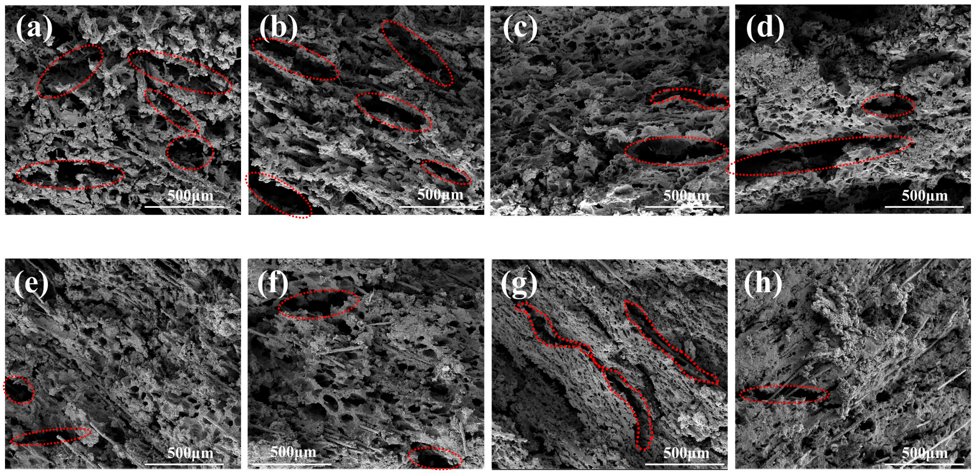

Figure S7. Effects of MWCNTs content on char layer cross-section of the EPDM-based insulators: NS-C-0 (a), NS-C-0.3 (b), NS-C-3 (c), NS-C-6 (d), S-C-0 (e), S-C-0.3 (f), S-C-3 (g) and S-C-6 (h).

8. Magnifying SEM image of char layer

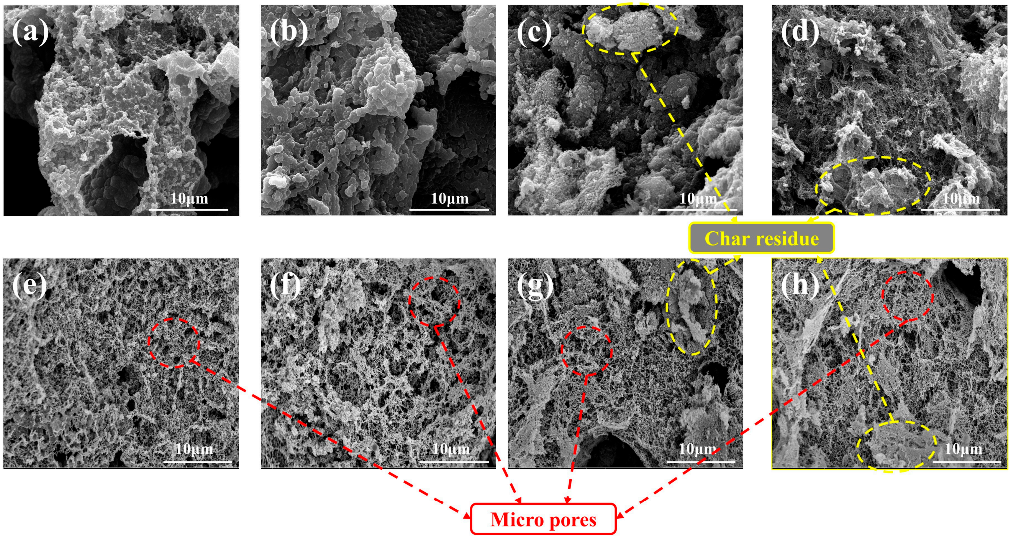

Figure S8. Effects of MWCNTs content on char layer surface of the EPDM-based insulators: NS-C-0 (a), NS-C-0.3 (b), NS-C-3 (c), NS-C-6 (d), S-C-0 (e), S-C-0.3 (f), S-C-3 (g) and S-C-6 (h).

Table S1. Formulations of EPDM-based insulators (phr[a])

Samples	EPDM (phr)	Silica (phr)	MWCNTs (phr)
S-C-0	100	20	0
S-C-0.3	100	20	0.3
S-C-0.7	100	20	0.7
S-C-1.5	100	20	1.5
S-C-3	100	20	3
S-C-6	100	20	6
NS-C-0	100	0	0
NS-C-0.3	100	0	0.3
NS-C-0.7	100	0	0.7
NS-C-1.5	100	0	1.5
NS-C-3	100	0	3
NS-C-6	100	0	6

AF: 10phr, Boron phenolic resin: 20phr, Sulphur: 0.5phr, BIPB: 4phr, TAIC: 2phr, zinc oxide: 5phr, stearic acid: 1phr, Paraffin oil: 4phr.

[a] phr: parts per hundred grams of EPDM.

Table S2. Effects of MWCNTs on mass ablation rate and linear ablation rate of EPDM-based insulators with and without silica.

Samples	Rm (g/s)	Rd (mm/s)	Rc (mm/s)
S-C-0	0.0396±0.0028	0.0980±0.0039	0.1635±0.0054
S-C-0.3	0.0480±0.0029	0.1029±0.0060	0.1807±0.0064
S-C-0.7	0.0508±0.0037	0.1076±0.0014	0.1778±0.0070
S-C-1.5	0.0412±0.0034	0.0973±0.0057	0.1656±0.0068
S-C-3	0.0389±0.0023	0.0880±0.0061	0.1559±0.0037
S-C-6	0.0427±0.0007	0.0837±0.0023	0.1519±0.0006
NS-C-0	0.0413±0.0011	0.1173±0.0051	0.1901±0.0087
NS-C-0.3	0.0413±0.0016	0.1092±0.0027	0.1911±0.0021
NS-C-0.7	0.0376±0.0019	0.1004±0.0039	0.1832±0.0065
NS-C-1.5	0.0402±0.0031	0.0904±0.0037	0.1790±0.0063
NS-C-3	0.0366±0.0007	0.0801±0.0016	0.1714±0.0053
NS-C-6	0.0390±0.0020	0.1011±0.0038	0.1720±0.0088

Table S3. Effects of MWCNTs content on thermal conductivity and back-face temperature (Tmax, b) of EPDM-based insulators with and without silica.

Samples	Back-face Temperature (°C)	Thermal Conductivity （W/mK）
S-C-0	84.5±3.6	0.233±0.003
S-C-0.3	86.8±3.1	0.238±0.004
S-C-0.7	87.4±2.3	0.243±0.003
S-C-1.5	98.0±6.7	0.258±0.002
S-C-3	99.1±3.7	0.272±0.003
S-C-6	106.1±3.8	0.304±0.006
NS-C-0	66.1±2.9	0.221±0.003
NS-C-0.3	68.0±4.0	0.220±0.001
NS-C-0.7	72.1±4.0	0.228±0.005
NS-C-1.5	78.9±5.4	0.235±0.003
NS-C-3	81.3±3.2	0.253±0.001
NS-C-6	97.9±2.1	0.289±0.002

Table S4. Effects of MWCNTs on mechanical property of EPDM-based insulators with and without silica

Samples	Tensile strength (Mpa)	Elongation at break (%)	Density (g/cm3)
S-C-0	5.51±0.41	147.8±26.0	1.035±0.002
S-C-0.3	5.79±0.22	182.5±12.1	1.040±0.001
S-C-0.7	5.89±0.16	211.6±24.6	1.042±0.001
S-C-1.5	5.98±0.33	284.6±14.8	1.051±0.002
S-C-3	6.07±0.25	310.5±31.1	1.058±0.005
S-C-6	6.31±0.14	362.8±43.7	1.064±0.002
NS-C-0	3.50±0.10	17.5±2.3	0.944±0.001
NS-C-0.3	3.77±0.31	10.6±0.8	0.951±0.001
NS-C-0.7	4.12±0.41	14.2±0.6	0.952±0.002
NS-C-1.5	5.02±0.18	16.4±1.8	0.960±0.004
NS-C-3	5.14±0.40	27.7±2.3	0.976±0.003
NS-C-6	5.31±0.21	49.5±5.3	0.994±0.001
